# A Guanine-Enhanced Graphene–DNA Paper-Based Sensing Platform Enabling Sensitive Hg^2+^ Detection

**DOI:** 10.3390/bios16040213

**Published:** 2026-04-10

**Authors:** Zihao Wu, Jingyan Li, Haixia Shi, Bing Xie, Li Gao

**Affiliations:** 1 School of Life Sciences, Jiangsu University, Zhenjiang 212013, China; 2212317017@stmail.ujs.edu.cn (Z.W.); 2212517025@stmail.ujs.edu.cn (J.L.); 2 P. E. Department, Jiangsu University, Zhenjiang 212013, China; 1000004860@ujs.edu.cn; 3 The Fourth Affiliated Hospital of Jiangsu University, Zhenjiang 212001, China; 4 School of Medicine, Jiangsu University, Zhenjiang 212013, China; 5 School of Life Sciences, Qinghai Normal University, Xining 810008, China

**Keywords:** biosensor, graphene, mercury ions, electrochemical detection, environmental monitoring

## Abstract

Mercury ions (Hg^2+^) are highly toxic and pose severe risks to human health and ecosystems, necessitating sensitive detection methods for environmental monitoring. Here, we report a paper-based graphene sensor functionalized with single-stranded DNA (ssDNA) probes for Hg^2+^ detection based on T-Hg^2+^-T coordination chemistry. To elucidate the effect of probe structure on sensing performance, we designed DNA constructs with varying numbers of guanine (G) bases (3–6, designated DNA2–DNA5) in the bridging fragment and systematically evaluated their influence on hairpin stability, Hg^2+^ binding affinity, and sensor response. The DNA3-based sensor (four G bases) exhibited optimal electronic stability and sensitivity, achieving a detection limit of 0.673 pM with effective real-time monitoring capability in aqueous media. These findings highlight the critical role of DNA sequence design in T-Hg^2+^-T-based biosensors and provide a promising strategy for sensitive and selective Hg^2+^ detection in environmental samples.

## 1. Introduction

Mercury, particularly in its ionic form (Hg^2+^), is recognized as one of the most hazardous heavy metal pollutants globally, presenting a persistent and serious threat to aquatic ecosystems and human health. Its toxicity is characterized by potent biological effects, as Hg^2+^ can enter the human body via drinking water or the aquatic food chain, causing irreversible damage to the central nervous system and kidneys. This may result in cognitive deficits, motor dysfunction, and even mortality. Consequently, mercury pollution has emerged as a major global public health and environmental concern, prompting stringent regulatory limits from national and international authorities. For instance, the United States Environmental Protection Agency has established a maximum contaminant level of 2 μg/L for mercury in drinking water and enforces strict controls on emissions [1]. Similarly, China classifies mercury as a priority pollutant under the “Environmental Quality Standards for Surface Water,” stipulating that its concentration in Class I surface water bodies must not exceed 0.05 μg/L.

In response to this critical need, various Hg^2+^ detection methods have been developed. Natural colorimetric sensors based on anthocyanin offer low cost and operational simplicity but suffer from high detection limits and pH dependence [2]. Nanocellulose-based fluorescent sensors achieve improved sensitivity yet involve complex synthesis procedures and exhibit temperature sensitivity [3]. A comparative summary of these representative approaches, along with graphene-based methods discussed below, is provided in Table 1. However, they also share common challenges, including the need for enhanced sensitivity to meet stricter regulatory standards, broader pH adaptability for complex environments, and more comprehensive field validation to confirm practical performance.

In contrast, sensors constructed using graphene as the transducer platform offer distinct advantages. These include exceptionally high charge carrier mobility, a large specific surface area for efficient biomolecule immobilization, excellent mechanical flexibility, and outstanding thermal and chemical stability [4]. When functionalized with appropriate recognition elements, graphene-based sensors achieve superior sensitivity, low limits of detection, rapid response times, and the potential for miniaturization and integration into portable devices [5].

Owing to its layered structure and sp^2^ hybridization characteristics, graphene offers carbon atoms that can be chemically modified with diverse functional groups tailored to specific biosensor performance requirements [6]. These modifications are facilitated by the adsorption sites provided by the π-electron system in graphene [7]. Its exceptionally high specific surface area enhances sensitivity to chemical and biological analytes [8]. Furthermore, the structural flexibility of graphene materials allows for surface engineering, which increases the density of recognition elements or analytical probes that can be immobilized on its surface [9]. Graphene and graphene-based materials can also be functionalized with various biological receptors through both covalent and non-covalent interactions [10,11]. As an ideal substrate for biosensing, graphene is widely employed in biosensors due to its high specific surface area and chemical versatility [12,13,14].

Researchers have developed several graphene–DNA sensors for mercury ion (Hg^2+^) detection. Building on these advantages, researchers have developed graphene-based Hg^2+^ sensors utilizing diverse transduction mechanisms. The key features of representative graphene-based Hg^2+^ sensors are summarized in Table 1. As shown, Xu et al. developed a fluorescence quenching platform based on graphene oxide and T-Hg^2+^-T coordination, achieving a detection limit of 0.5 nM [15]. Li et al. reported a field-effect transistor sensor incorporating an Al_2_O_3_ passivation layer, achieving 1 nM sensitivity with rapid response [16].

**Table 1:** Comparison of representative Hg^2+^ detection methods.

Method	LOD (pM)	Fabrication Complexity	Cost	Regeneration	Reference
Anthocyanin-based colorimetric sensor	6.48 × 10^7^	Low	Low	Not reported	[2]
Nanocellulose-based fluorometric sensor	4.11 × 10^4^	Moderate	Moderate	Yes	[3]
Graphene oxide-based fluorescence detection	1.10 × 10^5^	Moderate	Moderate	Not reported	[15]
Dual-mode SPR/SERS optical fiber sensor	0.394	High	High	Not reported	[16]
Pencil-drawn paper-based graphene–DNA	0.673	Low	Very low	Yes	This study

As shown in Table 1, while natural colorimetric and nanocellulose-based sensors offer advantages in cost and environmental friendliness, their sensitivity remains insufficient for trace-level detection. Graphene-based platforms achieve superior sensitivity but typically involve complex fabrication procedures, high material costs, and limited portability due to specialized instrumentation requirements. In contrast, the sensor developed in this work achieves a detection limit of 0.673 pM—comparable to or better than existing graphene-based sensors—while maintaining low fabrication complexity, minimal material cost, and inherent portability.

The development of such paper-based sensing platforms represents a broader trend in the field, addressing the limitations of conventional sensors through the unique advantages of paper substrates. In recent years, paper-based sensing platforms have evolved from simple single-function devices toward highly sensitive, multifunctional integrated systems capable of operating in complex environments. For instance, Lin et al. developed a photothermal–pyroelectric synergistic sensing platform based on a flexible polyimide–paper hybrid electrode, achieving ultrasensitive photoelectrochemical analysis of disease biomarkers under near-infrared excitation [17]. This work demonstrates that paper substrates, when combined with functional nanomaterials, can enable signal enhancement under complex physical field regulation, offering inspiration for further exploration of paper-based sensing platforms. Building on these insights, the fabrication and working principle of the pencil-drawn paper-based graphene–DNA sensor developed in this study are described in detail in the following section.

Building on these insights, the present study develops a paper-based graphene–DNA sensor fabricated via pencil drawing, which offers notable advantages in environmental sustainability, low cost, and ease of preparation [18,19]. As highlighted by Zhang et al. in their comprehensive review on multifunctional cellulose paper-based materials, paper substrates—owing to their abundant hydroxyl groups, porous three-dimensional network structure, and favorable biocompatibility—can be functionalized through various chemical or physical modification strategies, demonstrating great potential in sensing applications [20]. These properties provide a solid material foundation for the development of paper-based mercury ion sensors.

A key advantage of the proposed fabrication method lies in the use of pencil graphite as the graphene source. In contrast to conventional approaches such as chemical vapor deposition and mechanical exfoliation, this strategy incurs negligible cost, offers high safety, and provides an efficient, environmentally friendly acquisition route [21,22]. Li et al. demonstrated that pencil traces drawn on paper consist of percolated networks of fine graphite particles, which inherently contain graphene-like structures and exhibit reversible resistance changes upon mechanical deformation or chemical exposure, confirming the presence of graphene-based materials in pencil marks [23]. This work establishes pencil drawing as a simple, rapid, and effective method for creating functional conductive pathways on paper substrates. Xiong et al. adopted a similar approach when fabricating flexible paper-based supercapacitors, demonstrating that a uniform conductive graphite layer can be formed on paper by simply drawing with a 2B pencil, which serves as an ideal substrate for subsequent functionalization [24]. This confirms that pencil drawing is a simple, rapid, and effective technique for constructing conductive pathways on paper substrates.

Specific detection of Hg^2+^ was achieved by integrating a Schiff base reaction scheme into the graphene–DNA sensing platform. This approach exploits the formation of a stable T-Hg^2+^-T complex between Hg^2+^ and the thymine–thymine (T-T) mismatch within the DNA probe sequence [25,26]. Based on this principle, the paper-based sensor was designed using standard A4 printing paper as the substrate, with electrode channels fabricated via pencil-deposited graphite. The condenser agent 1,5-diaminonaphthalene (DAN) was immobilized on the graphite/graphene surface through π-π stacking interactions between its pyrenyl group and the graphitic plane [27]. Glutaraldehyde (GA) was then conjugated to DAN via a Schiff base reaction, followed by the immobilization of amino-modified DNA onto the aldehyde groups of GA through a second Schiff base linkage. This stepwise functionalization process yielded the completed graphene–DNA sensor [28,29,30]. The covalent coupling strategy employed here is consistent with the design principles summarized by Wang et al. [31] in their review on paper-based optical detection devices, which emphasizes that Schiff base reactions and other covalent bonding strategies can effectively enhance the stability and uniformity of immobilized recognition elements, thereby improving overall detection performance. The binding of Hg^2+^ induces a measurable change in electrical signal, enabling quantitative detection.

Guanine-rich (G-rich) DNA sequences can fold into non-canonical secondary structures known as G-quadruplexes, which are stabilized by Hoogsteen hydrogen bonding. These structures play significant roles in gene regulation and chemical biosensing [32]. Their formation and stability are strongly influenced by metal cations, with Hg^2+^ known to enhance G-quadruplex stability under certain conditions [33]. To systematically investigate the effect of G-quadruplex stability on T-Hg^2+^-T recognition efficiency, a series of DNA probes containing 3, 4, 5, and 6 consecutive guanine bases (denoted as 3G–6G) were designed.

The selection of this specific range was guided by established principles of G-quadruplex formation. Stable intramolecular folding generally requires a minimum of three consecutive guanines in each G-tract to form a G-tetrad core, sequences with fewer than three contiguous guanines are typically unable to adopt stable G-quadruplex structures under physiological conditions [34,35]. Conversely, tracts longer than six consecutive guanines tend to form highly stable intermolecular aggregates or higher-order structures (e.g., G-wires), which can introduce non-specific interactions and compromise sensor reproducibility [36]. Thus, the selected range of 3–6 consecutive guanines span the transition from minimally stable to highly stable G-quadruplex structures while preserving intramolecular folding and sequence solubility, enabling a systematic investigation of the relationship between G-quadruplex stability and T-Hg^2+^-T recognition efficiency.

This investigation aims to provide crucial design principles and a theoretical foundation for the future development of high-performance, multifunctional DNA-based nanodevices and sensors. Ultimately, this study underscores the potential of the developed paper-based graphene–DNA sensor for applications in environmental monitoring and related fields.

## 2. Materials and Methods

### 2.1. Materials

All DNA samples:

DNA1: 5′-NH_2_-CCACCACTTTTTTTTTGGTTTTTTTTT-3′;
DNA2: 5′-NH_2_-CCACCACTTTTTTTTTGGGTTTTTTTTT-3′;
DNA3: 5′-NH_2_-CCACCACTTTTTTTTTGGGGTTTTTTTTT-3′;
DNA4: 5′-NH_2_-CCACCACTTTTTTTTTGGGGGTTTTTTTTT-3′;
DNA5: 5′-NH_2_-CCACCACTTTTTTTTTGGGGGGTTTTTTTTT-3′;
DNA6: 5′-NH_2_-CCACCACAAAAAAAAAGGGGTTTTTTTTT-3′;
DNA7: 5′-NH_2_-CCACCACACTACTACTGGGGAGTAGTAGT-3′.

These samples and 1,5-diaminonaphthalene (DAN), 25% glutaraldehyde (GA) were purchased from Sangon Biotech (Shanghai) Co., Ltd. (Shanghai, China). Methyl alcohol was purchased from Yonghua Chemical Co., Ltd. (Changshu, China). 9B pencils were purchased from Hebei Chinjoo Art Materials Technology Co., Ltd. (Hengshui, China). The A4 papers were purchased from Shanghai M&G Stationery Inc (Shanghai, China). Mercuric nitrate (Hg(NO_3_)_2_), sodium nitrate (NaNO_3_), potassium chloride (KCl), zinc chloride (ZnCl_2_), magnesium chloride (MgCl_2_·6H_2_O), calcium chloride (CaCl_2_·2H_2_O), lead acetate (Pb(CH_3_COO)_2_·_3_H_2_O), copper sulfate (CuSO_4_·_5_H_2_O), manganese chloride (MnCl_2_·4H_2_O), hydrochloric acid (HCl) and sodium hydroxide (NaOH) were purchased from Sinopharm Chemical Reagent Co., Ltd. (Shanghai, China). Dithiothreitol (DTT) was purchased from Jiangsu Centron Biotechnology Co., Ltd. (Taizhou, China).

### 2.2. Methods

#### 2.2.1. The Fabrication of Sensors

A4 printing paper was employed as the substrate material. A conductive channel was fabricated on the paper using a 9B pencil, with the two termini defined as the source and drain electrodes (Figure S1), and the intermediate segment functioning as the sensing channel. Scanning electron microscopy (SEM) imaging confirmed the successful formation of graphitic material on the drawn line (Figure S2). The paper-based graphitic film was further characterized by laser micro-Raman spectroscopy. The Raman spectrum (Figure S3) exhibits characteristic peaks, including the 2D peak, which corresponds to second-order zone-boundary phonons, and the G peak, originating from the in-plane vibrational mode of sp^2^-hybridized carbon atoms. The position of the G peak serves as an indicator for estimating the number of graphene layers. Raman spectroscopy was performed to characterize the pencil-drawn graphite layer. As shown in Figure S3, the spectrum exhibits characteristic D and G bands at approximately 1350 cm^−1^ and 1580 cm^−1^, respectively, confirming the presence of multilayer graphene. The ID/IG ratio was calculated to be 0.084, indicating a low defect density in the pencil-drawn graphite layer. This high crystalline quality provides a well-ordered π-conjugated surface favorable for π-π stacking immobilization of 1,5-diaminonaphthalene (DAN). To evaluate the uniformity and reproducibility of the pencil-drawn graphite layer, sheet resistance was measured at five positions along three independently prepared channels (Figure S4). The results showed consistent sheet resistance across all samples, with coefficients of variation below 10% for each channel, confirming the reliability of the pencil-drawing method.

Subsequently, 40 µL of a 15 µM methanolic solution of 1,5-diaminonaphthalene (DAN) was applied to the graphene channel and allowed to react at room temperature for 3 h, after which the substrate was dried. Following this, 40 µL of a 2% glutaraldehyde (GA) solution was introduced onto the electrode surface and reacted under the same conditions for an additional 3 h, followed by drying. In the final functionalization step, 40 µL of each DNA probe solution at a concentration of 40 µM was deposited onto the respective electrodes. The samples were then incubated at 4 °C for 6 h and subsequently dried.

The successful stepwise conjugation was verified by Fourier transform infrared (FT-IR) spectroscopy (Figure S5). Characteristic absorption peaks corresponding to key functional groups were observed. Specifically, the N–H stretching vibration at 3371 cm^−1^ and the C=O stretching vibration at 1715 cm^−1^ were identified. The presence of a C–H stretching band at 2934 cm^−1^ and a C=N stretching vibration at 1600 cm^−1^ con-firmed the successful linkage of DAN to GA and the subsequent immobilization of DNA via Schiff base formation. Furthermore, the morphology of the functionalized graphene was examined by scanning electron microscopy (SEM), as shown in Figure S6.

#### 2.2.2. Optimization of Detection Conditions for Paper-Based Graphene–DNA Sensors

The experimental conditions were systematically optimized, including the effect of ultrapure (UP) water (Figure S7), as well as with respect to detection frequency (Figure S8), applied voltage (Figure S9), DAN concentration (Figure S10), DNA probe concentration (Figure S11), Hg^2+^ reaction time (Figure S12), reaction temperature (Figure S13) and the pH value of the reaction (Figure S14).

#### 2.2.3. Hg^2+^ Sensitivity Detection with Graphene–DNA Sensors

The baseline current was first acquired using a lock-in amplifier. Subsequently, 30 µL of Hg^2+^ solution at varying concentrations was introduced onto the functionalized graphene–DNA sensor surface and allowed to react at room temperature for 1 h. The resulting change in current was then measured with the lock-in amplifier to assess the sensor response.

#### 2.2.4. Hg^2+^ Selectivity Detection with Graphene–DNA Sensors

The baseline current was first recorded using a lock-in amplifier. Subsequently, 30 µL of solutions containing different metal ions—specifically Pb^2+^, Na^+^, Mg^2+^, Cu^2+^, Mn^2+^, K^+^, Zn^2+^, Ca^2+^, and Hg^2+^—were separately introduced onto the surface of the prepared graphene–DNA sensors. Each sensor was incubated with the respective ion solution at room temperature for 1 h. The corresponding change in current was then measured with the lock-in amplifier to evaluate and compare the sensor’s response, thereby assessing its selectivity toward Hg^2+^ over potentially interfering ions.

#### 2.2.5. Regeneration Cycle Experiment of Graphene–DNA Sensor

Dithiothreitol (DTT), a dithiol compound commonly employed as a small-molecule reducing agent, contains terminal sulfhydryl groups at both ends. The strong affinity of sulfhydryl groups for Hg^2+^ arises from the low electronegativity and high polarizability of sulfur, coupled with the relatively large ionic radius and low charge density of the Hg^2+^ ion. DTT can interact with Hg^2+^ via a reversible complexation reaction. This property was utilized to confirm the successful formation of the T-Hg^2+^-T complex within the multi-thymine “hairpin” structure of the DNA probe.

The initial current (I_0_) of the fabricated graphene–DNA sensor was measured using a lock-in amplifier. Subsequently, 30 μL of a 1000 pM Hg^2+^ solution was applied to the sensor surface and allowed to incubate at room temperature for 1 h, after which the resultant current was recorded. To regenerate the sensor, 30 μL of a 2.5 mM dithiothreitol (DTT) solution was then introduced, followed by a 1 h incubation period at 4 °C, and the stabilized current (I) was measured. The sensor surface was then gently rinsed with 1 mL of ultrapure water. This complete cycle—Hg^2+^ binding, measurement, DTT regeneration, and rinsing—was repeated to assess the sensor’s reusability.

#### 2.2.6. Control Experiment for Mechanism Validation

To confirm that the sensor response is specifically mediated by T-Hg^2+^-T coordination, two control DNA probes were designed. The first control probe (DNA6) retains the same hairpin framework as the original probe but replaces the T-T mismatch region with A-T Watson–Crick base pairs, thereby eliminating the thymine–thymine mismatches required for T-Hg^2+^-T formation. The second control probe (DNA7) contains a randomized sequence in the recognition region that lacks both T-T mismatches and any known metal-binding motifs, while maintaining a similar secondary structure framework.

The initial current (I_0_) of the fabricated graphene–DNA sensor was measured using a lock-in amplifier. Subsequently, 30 μL of a 1000 pM Hg^2+^ solution was applied to the sensor surface and allowed to incubate at room temperature for 1 h, after which the resultant current was recorded. The responses were then compared with those obtained from the original sensor to validate the contribution of the T-Hg^2+^-T coordination to the sensor response.

## 3. Results

### 3.1. Sensitivity Analyses of Different Number of G Base

To systematically investigate the influence of guanine (G) base count on hairpin stability, Hg^2+^ binding efficiency, and overall sensor performance, paper-based graphene–DNA sensors were fabricated using the designed probes DNA2, DNA3, DNA4, and DNA5, containing 3, 4, 5, and 6 contiguous G bases, respectively. The sensitivity of each sensor variant toward Hg^2+^ was evaluated via electrical measurements using a lock-in amplifier. A constant source–drain bias voltage of 100.0 mV with zero gate bias was applied at a frequency of 35.0 kHz. The baseline current (I_0_) for each sensor was first recorded under these conditions. Subsequently, droplets of Hg^2+^ solutions at varying concentrations were applied to the sensor surfaces. Following a 1 h incubation period at a temperature of 20 °C to allow for probe–ion interaction, the current was measured again under identical bias conditions.

**Figure 1:**
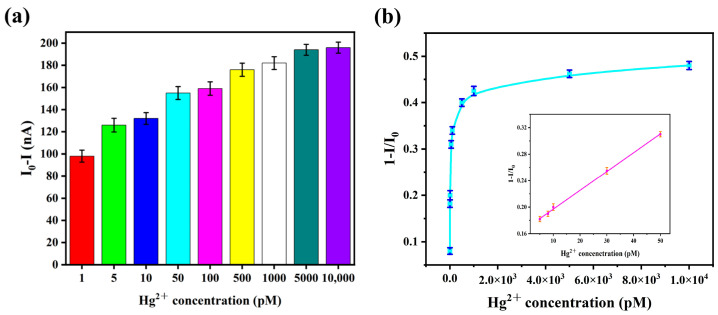
(**a**) Sensitivity analysis of Hg^2+^ based on graphene–DNA3 sensors. (**b**) Current inhibition rate as a function of mercury ion concentration based on graphene–DNA3 sensors.

The resultant calibration data are presented in Figure S15 (3G), Figure 1 (4G), Figure S16 (5G), and Figure S17 (6G). The calculated limits of detection (LOD) for the DNA2-, DNA3-, DNA4-, and DNA5-based sensors were 0.875 pM, 0.673 pM, 2.66 pM, and 3.78 pM, respectively. Among the four probe configurations, the sensor functionalized with DNA3 (containing 4G bases) demonstrated the highest sensitivity and the lowest detection limit. This result indicates that the DNA3 sequence provides the most effective performance in transducing Hg^2+^ binding into an enhanced sensor response.

When the concentration of Hg^2+^ was in the range of 5–50 pM, there was a clear linear relationship between the current change and it. The linear equation was y = 0.003x + 0.1682, R^2^ = 0.996, and the detection limit was 0.673 pM. Detailed calculation of the limit of detection (LOD) is presented in Section S4 of the Supplementary Materials. To verify the successful binding of mercury ions to DNA, we used a UV spectrophotometer for characterization. When analyzed by ultraviolet spectrophotometry, each exhibits a characteristic absorption maximum at a wavelength of 260 nm. Upon addition of Hg^2+^, the thymine (T) bases selectively coordinate with the metal ion to form a T-Hg^2+^-T complex. This metal-mediated base pairing induces a transition towards a duplex-like structure, resulting in a measurable decrease in the absorbance at 260 nm due to hypochromicity. The result of this analysis is presented in Figure S18 (for the 4G construct). The absorption peak at 260 nanometers has significantly decreased, leading to the most effective formation of the T-Hg^2+^-T complex.

### 3.2. Selectivity Analyses of Graphene–DNA3 Sensor

**Figure 2:**
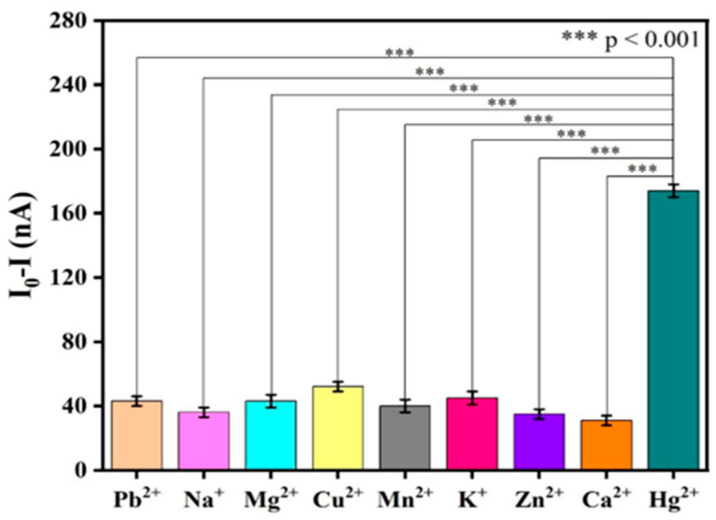
Detection of different metal ions based on graphene–DNA3 sensors.

The selectivity of the graphene–DNA sensors functionalized with DNA3, containing 4 guanine (G) bases, respectively, was evaluated against Hg^2+^. To assess specificity, each sensor was exposed individually to various metal ions (e.g., Pb^2+^, Na^+^, Mg^2+^, Cu^2+^, Mn^2+^, K^+^, Zn^2+^, and Ca^2+^) at an identical concentration of 1000 pM. Following incubation at a temperature of 20 °C for 1 h, the corresponding current response was measured using a lock-in amplifier. The result is presented in Figure 2. The introduction of Hg^2+^ induced a substantially greater current decrease compared to any of the other tested metal ions, confirming the specific interaction between Hg^2+^ and the thymine bases within the DNA probes. Statistical analysis verified that the response to Hg^2+^ was significantly distinct from the responses to interfering ions, thereby further validating the sensor’s high selectivity.

**Figure 3:**
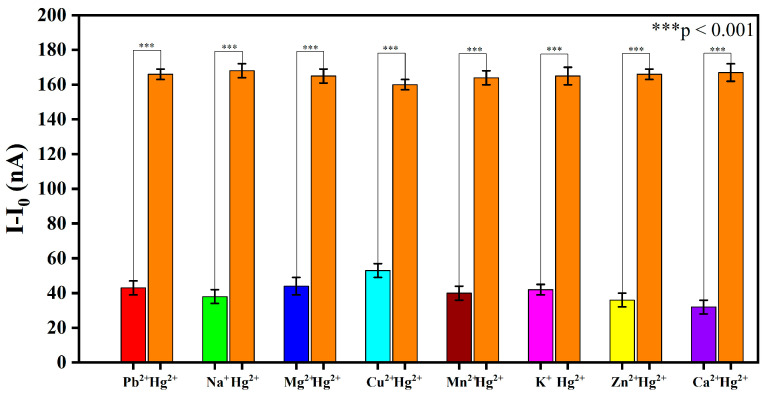
Detection of cross-interference between mercury ions and another metal ion based on graphene–DNA3 sensors.

To evaluate potential cross-interference, graphene–DNA3 sensors were employed to investigate the sensor’s response in the presence of co-existing metal ions. Initially, other metal ions (e.g., Pb^2+^, Cu^2+^, Zn^2+^, etc.) at an equal concentration of 1000 pM were individually introduced onto the sensor surface and incubated at room temperature for 1 h, and the corresponding current response was measured using a lock-in amplifier. Subsequently, 1000 pM of Hg^2+^ was added to the same sensor (without removal of the pre-existing ion) and allowed to react for an additional hour under identical conditions, after which the current was measured again. The results, presented in Figure 3, demonstrate that the current shift induced by the subsequent addition of Hg^2+^ was significantly greater than the response caused by any of the other individual ions alone. Statistical analysis confirmed that the response to Hg^2+^ remained pronounced and distinct even in the presence of potential interferents, further validating the high selectivity of the DNA3-based sensor for Hg^2+^ detection.

The introduction of Hg^2+^ resulted in a substantially greater decrease in current relative to other metal ions, confirming a specific and strong interaction between Hg^2+^ and the thymine-rich DNA sequences. This selectivity arises from the T-Hg^2+^-T coordination, a well-established DNA-based recognition mechanism for mercury ions. In standard Watson–Crick base pairing, thymine (T) forms hydrogen bonds with adenine (A). However, in the presence of Hg^2+^—a “soft” Lewis acid with high affinity for nitrogen and sulfur donors—the ion selectively coordinates to the N_3_ atoms of two opposing thymine bases. This forms a stable, linear T-Hg^2+^-T complex through metal-mediated bonds, effectively pulling the two thymines together into a mismatched pair that is more stable than the natural A-T pairing under the given conditions.

To better simulate real-world environmental conditions, a competition-mode experiment was performed in which Hg^2+^ (1000 pM) was mixed with all eight interfering metal ions (Pb^2+^, Na^+^, Mg^2+^, Cu^2+^, Mn^2+^, K^+^, Zn^2+^, Ca^2+^), each at the same concentration of 1000 pM.

**Figure 4:**
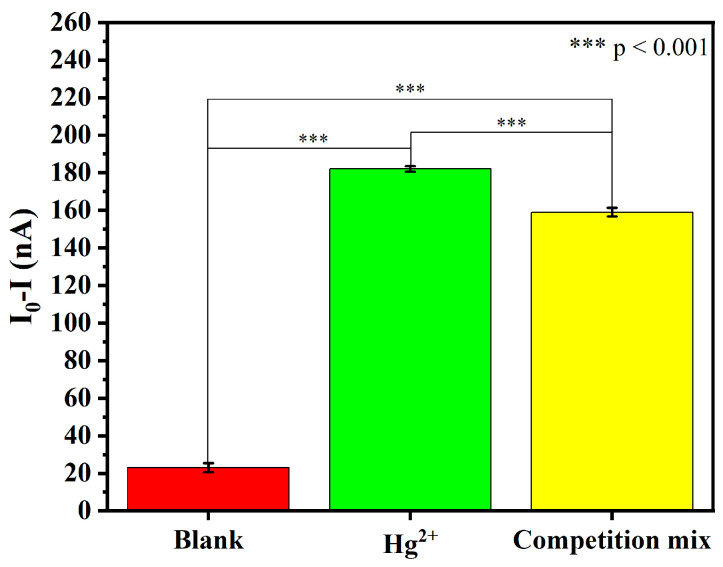
Sensor response to Hg^2+^ alone and in competition with interfering metal ions.

As shown in Figure 4, the sensor exhibited a strong and clearly detectable response to Hg^2+^ alone. In the presence of a mixture of interfering metal ions, the sensor’s response remained substantially higher than the blank control, although a slight decrease was observed. Statistical analysis revealed a significant difference between the Hg^2+^ alone and competition groups; nevertheless, the sensor retained the majority of its original signal under competitive conditions. These results demonstrate that the sensor maintains excellent selectivity for Hg^2+^ even in the presence of high concentrations of competing metal ions, confirming its suitability for real-world environmental water analysis.

### 3.3. Regeneration Cycle Experiment of Graphene–DNA3 Sensor

**Figure 5:**
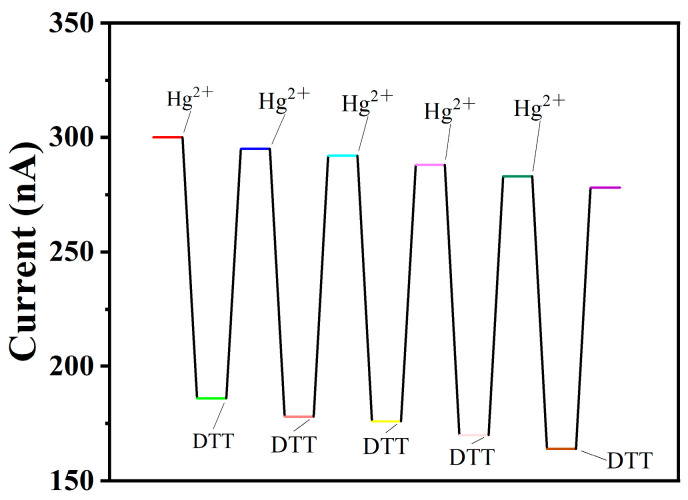
Regeneration cycle stability of graphene–DNA3 sensor.

The regeneration cycle stability of the paper-based graphene–DNA3 sensor was systematically evaluated, with the results summarized in Figure 5.

The regeneration mechanism relies on the competitive binding affinity between the sensor’s DNA probe and dithiothreitol (DTT) for Hg^2+^. The thiol groups in DTT exhibit strong, reversible coordination with Hg^2+^ due to soft acid–soft base interactions. When DTT is introduced, it effectively sequesters Hg^2+^ from the T–Hg^2+^–T complex, disrupting the metal-mediated base pair. This releases Hg^2+^ from the DNA structure, allowing the probe to revert to its original conformation and restoring the baseline electron transport properties of the graphene interface. This reversible process enables multiple detection–regeneration cycles without significant loss of sensor performance. Upon reintroduction of Hg^2+^ to the sensor surface following DTT regeneration, a significant decrease in current was again observed. This confirms that Hg^2+^ competitively binds to the thiol groups made available by DTT, effectively reversing the sequestration process.

### 3.4. Control Experiments for T-Hg^2+^-T Recognition

To confirm that the observed sensor response originates specifically from T-Hg^2+^-T coordination rather than from non-specific interactions or the presence of a hairpin structure alone, two control DNA probes were tested. The first control probe (DNA6) replaces the T-T mismatch region with A-T Watson–Crick base pairs. The second control probe (DNA7) contains a randomized recognition sequence that lacks both T-T mismatches and any known metal-binding motifs, while maintaining a similar secondary structure framework.

**Figure 6:**
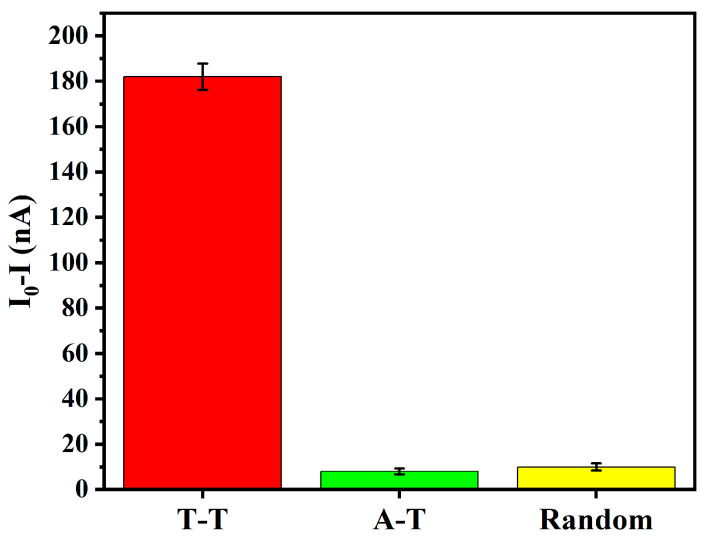
Control experiment for T-Hg^2+^-T recognition mechanism.

As shown in Figure 6, the sensor using the T-T probe exhibited a strong and clearly detectable response to Hg^2+^. In contrast, both the A-T and Random control probes showed only negligible current changes under identical conditions. These results confirm that the observed sensor response is specifically mediated by T-Hg^2+^-T coordination rather than non-specific interactions or the presence of a hairpin structure alone.

### 3.5. Testing in Real Samples

To practically evaluate the graphene–DNA3 sensor, it was used to determine Hg^2+^ in ultrapure water using standard addition methods. Known concentrations of Hg^2+^ (10 pM, 50 pM, and 100 pM) were spiked into the filtered samples for recovery tests. The results are summarized in Table 2. The average recovery rates ranged from 97.6% to 103.4%, with relative standard deviations (RSD) between 1.6% and 3.2%, demonstrating satisfactory accuracy and reproducibility.

To assess the practical performance of the paper-based graphene–DNA3 sensor, Hg^2+^ detection was carried out in real environmental water samples, including water collected from the Yangtze River, tap water, and drinking water. Prior to analysis, each sample was filtered through a 0.22 μm membrane to remove particulate impurities. The results are summarized in Table 3.

The concentration of Hg^2+^ detected by graphene–DNA3 sensors are all below the standard value, conforms to the experimental expectations. These findings confirm that the proposed sensor offers reliable and sensitive detection of Hg^2+^, fulfilling the practical requirements for environmental monitoring applications

## 4. Discussion

### 4.1. Regulatory Mechanism: Modulation of T-Hg^2+^-T Formation by the Adjacent G-Quadruplex

To investigate the regulatory mechanism by which the adjacent G-quadruplex influences T-Hg^2+^-T recognition efficiency, a series of DNA probes containing 3, 4, 5, and 6 consecutive guanine bases (denoted as 3G–6G) were designed for systematic comparison. Experimental results revealed a non-monotonic trend in detection performance with increasing G-quadruplex length, suggesting that the G-quadruplex modulates T-Hg^2+^-T formation primarily through conformational regulation and base accessibility.

As a rigid, compact secondary structure, the formation of a G-quadruplex significantly alters the local conformation and flexibility of the adjacent T-rich region. With increasing G-quadruplex length, its thermodynamic stability enhances, potentially locking the probe in a specific conformation and thereby affecting the accessibility of thymine bases to Hg^2+^. For probes with shorter G-tracts (3G), the G-quadruplex may be insufficiently stable to pre-organize the T-rich region effectively. In contrast, probes with longer G-tracts (5G, 6G) may exhibit excessive stability, over-constraining the adjacent region and hindering the conformational flexibility required for efficient T-Hg^2+^-T coordination. Among the probes tested, the probe containing four consecutive guanines (4G) exhibited optimal recognition efficiency, likely due to an optimal conformational balance between G-quadruplex stability and the flexibility of the T-rich region.

In summary, through systematic variation in G-quadruplex length, this study establishes a quantitative relationship between structural stability and recognition efficiency. The 4G probe achieved an optimal balance between conformational stability and base accessibility, resulting in the best Hg^2+^ detection performance. This finding provides important theoretical guidance for the optimized design of DNA-based sensors in future applications.

### 4.2. Key Metrics Comparison of Recent Graphene-Based Hg^2+^ Sensors

Numerous studies have focused on the development of graphene-based DNA sensors for the detection of mercury ions, aiming to achieve high sensitivity and selectivity. To realize more efficient, stable, and user-friendly sensing platforms, continued refinement in design and fabrication is essential. Consequently, the exploration of novel approaches remains an important research objective in this field. Table 4 summarizes the latest developments in graphene–DNA Hg^2+^ biosensors.

## 5. Conclusions

In conclusion, this study successfully developed a low-cost, user-friendly paper-based graphene–DNA sensor for the highly sensitive and selective detection of mercury ions. Systematic evaluation of DNA probes containing varying numbers of guanine (G) bases revealed that the DNA3-functionalized sensor, incorporating four consecutive G bases, delivered optimal performance. It achieved a detection limit as low as 0.673 pM and exhibited superior selectivity for Hg^2+^, effectively discriminating against potential interference from other common metal ions. The sensor maintained reliable operation across a temperature range of 20–50 °C and a pH window of 6–10, demonstrating good environmental adaptability. Furthermore, regeneration studies confirmed that the sensor could undergo multiple detection–recovery cycles with minimal performance degradation, underscoring its reusability and practical economy. Validation using real environmental water samples yielded satisfactory recovery rates (97.6–103.4%) and low relative standard deviations (1.6–3.2%), confirming its accuracy, reproducibility, and suitability for real-world applications. Collectively, this work presents a high-performing, practical sensing platform with significant potential for monitoring trace-level Hg^2+^ contamination in aquatic environments, offering a viable and effective solution for environmental water quality assessment.

## Figures and Tables

**Table 2:** The recovery rates of standard addition at different concentration levels (*n* = 3).

Samples	Added (pM)	Average Recovery (%)	SD (%)	RSD (%)
Ultrapure water	10	97.6	3.1	3.2
Ultrapure water	50	98.3	2.4	2.4
Ultrapure water	100	103.4	1.7	1.6

**Table 3:** Analysis of mercury ions in actual samples (*n* = 3).

Sample	Hg^2+^ Concentration Standard (pM)	Determination of Hg^2+^ (pM)
Yangtze River	<5	0.96 ± 0.03
Tap Water	<5	0
Drinking Water	<5	0

**Table 4:** Key metrics comparison table of recent graphene-based Hg^2+^ sensors.

LOD (pM)	Range (pM)	Linearity	Selectivity	Reference
5.0 × 10^2^	2.5 × 10^3^–4.0 × 10^4^	R^2^ = 0.99	High selectivity	[37]
1.4 × 10^3^	0–2.0 × 10^5^	R^2^ = 0.99	High selectivity	[38]
1.5 × 10^2^	0–1.0 × 10^5^	R^2^ = 0.97	High selectivity	[39]
5.0 × 10^1^	5.0 × 10^1^–3.3 × 10^3^	R^2^ = 0.94	High selectivity	[40]
5.0 × 10^2^	1.0 × 10^3^–2.0 × 10^6^	R^2^ = 0.99	High selectivity	[41]
4.5 × 10^1^	1.0 × 10^3^–1.0 × 10^7^	R^2^ = 0.99	High selectivity	[42]
1.3 × 10^5^	1.6 × 10^6^–1.4 × 10^8^	R^2^ = 0.99	High selectivity	[43]
1.7 × 10^6^	1.0 × 10^6^–1.0 × 10^7^	R^2^ = 0.99	High selectivity	[44]
9.3 × 10^4^	6.0 × 10^4^–6.0 × 10^5^	R^2^ = 0.99	High selectivity	[45]
5.2	2.0 × 10^4^–2.0 × 10^9^	R^2^ = 0.99	High selectivity	[46]
0.7	5.0–5.0 × 10^1^	R^2^ = 0.99	High selectivity	This study

## Data Availability

The original contributions presented in this study are included in the article/Supplementary Materials. Further inquiries can be directed to the corresponding authors.

## Supplementary Materials

The following supporting information can be downloaded at: https://www.mdpi.com/article/10.3390/bios16040213/s1, Figure S1: Graphene sensor based on paper; Figure S2: Image of graphene under scanning electron microscopy. (a) SEM images of the pencil-drawn graphite channel at 10 µm scale. (b) SEM images of the pencil-drawn graphite channel at 5 µm scale; Figure S3: Raman spectra of graphene; Figure S4: Sheet resistance (*R_s_*) of three independently prepared pencil-drawn graphite channels (Sample 1–3); Figure S5: Infrared spectra of DAN, DAN+GA, and DAN+GA+DNA; Figure S6: Image of functionalized graphene under a scanning electron microscope. (a) SEM images of the pencil-drawn graphite channel after functionalization at 10 µm scale. (b) SEM images of the pencil-drawn graphite channel after functionalization at 5 µm scale; Figure S7: Effect of UP water on the conductivity of the graphene sensor; Figure S8: Signal-to-noise ratio with different frequencies; Figure S9: Current under different voltages between source and drain; Figure S10: Optimization results of DAN concentration; Figure S11: Optimization results of DNA1 concentration; Figure S12: Optimization results of Hg^2+^ reaction time; Figure S13: Responses of graphene-DNA1 sensor at different temperatures; Figure S14: Responses of graphene-DNA1 sensor at different pH value; Figure S15: (a) Sensitivity analyses of Hg^2+^ based on graphene-DNA2 sensors. (b) Current inhibition rate as a function of Hg^2+^ concentration based on graphene-DNA2 sensors; Figure S16: (a) Sensitivity analyses of Hg^2+^ based on graphene-DNA4 sensors. (b) Current inhibition rate as a function of Hg^2+^ concentration based on graphene-DNA4 sensors; Figure S17: (a) Sensitivity analyses of Hg^2+^ based on graphene-DNA5 sensors. (b) Current inhibition rate as a function of Hg^2+^ concentration based on graphene-DNA5 sensors; Figure S18: Absorbance of DNA3 and DNA3 with Hg^2+^.
