# Supplementary material for: A Guanine-Enhanced Graphene–DNA Paper-Based Sensing Platform Enabling Sensitive Hg2+ Detection
Source: Biosensors (Basel). 2026 Apr 10;16(4):213. doi: 10.3390/bios16040213 (PMC13113891; doi:10.3390/bios16040213)
Supplement: Supplementary file 1 [file biosensors-16-00213-s001.zip › biosensors-4232588-supplementary.pdf]

biosensors

Supplementary Material

S1. Characterization of graphene sensor

S1.1 Photo of graphene sensor

Figure S1 shows the prepared paper-based graphene sensor.

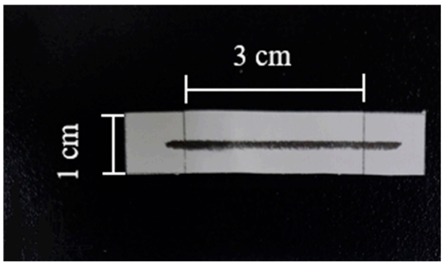

Figure S1. Graphene sensor based on paper.

S1.2 Scanning electron microscope image of graphene sensors

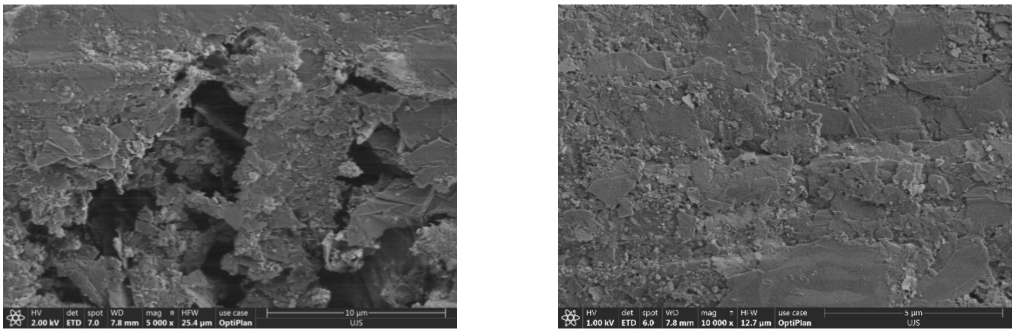

(**a**) (**b**)

Figure S2. Image of graphene under scanning electron microscopy. (**a**) SEM images of the pencil-drawn graphite channel at 10 μm scale. (**b**) SEM images of the pencil-drawn graphite channel at 5 μm scale.

S1.3 Infrared characterization of graphene and DNA connection

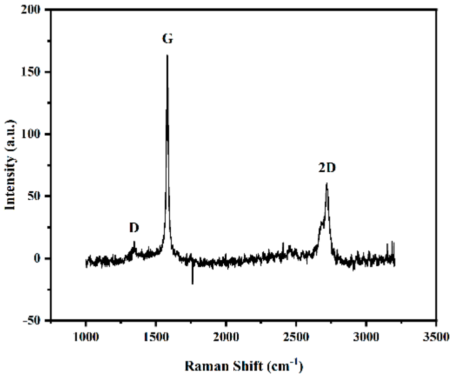

Figure S3. Raman spectra of graphene.

S1.4 Sheet resistance of pencil-drawn graphite channels

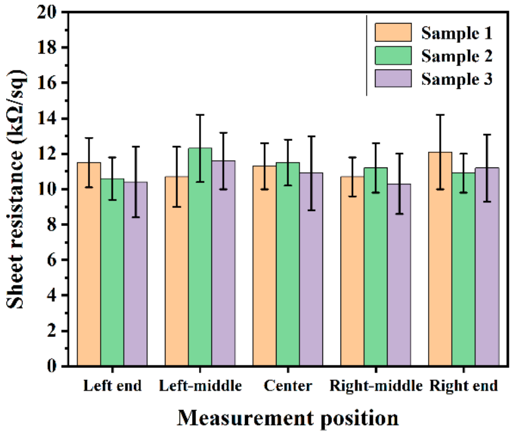

Figure S4. Sheet resistance (R_s) of three independently prepared pencil-drawn graphite channels (Sample 1–3).

Each channel was divided into five equal segments (7 mm each) along its length (35 mm). The sheet resistance was calculated for each segment from the measured resistance using the formula $R_S = R_{measure} \times (W/L_{seg})$, where W=2 mm and L_{seg} =7 mm. The data are presented as the mean of five repeated measurements per segment.

To quantitatively evaluate the electrical uniformity along each pencil-drawn channel, the coefficient of variation (CV) was calculated for the sheet resistance values measured at five different positions. CV provides a dimensionless measure of relative variability, allowing direct comparison of dispersion across samples regardless of the absolute sheet resistance magnitude.

$$CV = \left(\frac{\sigma}{\bar{x}}\right) \times 100\% \quad (1)$$

σ = Standard deviation of the five-segment sheet resistances;

$\bar{x}$ = Mean of the five segment sheet resistances.

For Sample 1, the sheet resistance values gave a mean of 11.26 kΩ/sq and a standard deviation of 0.59 kΩ/sq, resulting in a CV of 5.2%. Sample 2 showed a mean of 11.30 kΩ/sq, a standard deviation of 0.65 kΩ/sq, and a CV of 5.8%. Sample 3 exhibited a mean of 10.88 kΩ/sq, a standard deviation of 0.54 kΩ/sq, and a CV of 5.0%. All three samples yielded CV values below 6%, indicating excellent electrical uniformity along each channel.

S2. Characterization of functionalized graphene sensors

S2.1 Infrared characterization of graphene and DNA connection

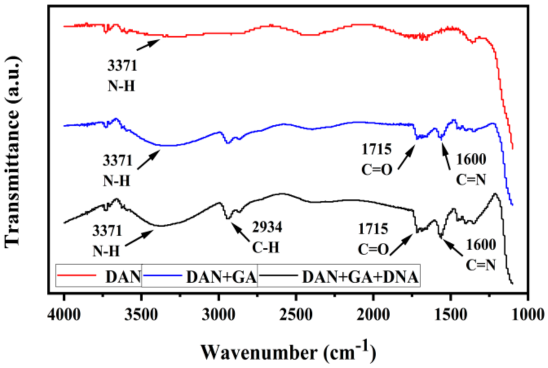

Figure S5. Infrared spectra of DAN, DAN+GA, and DAN+GA+DNA.

S2.2 Scanning electron microscope image of functionalized graphene sensors

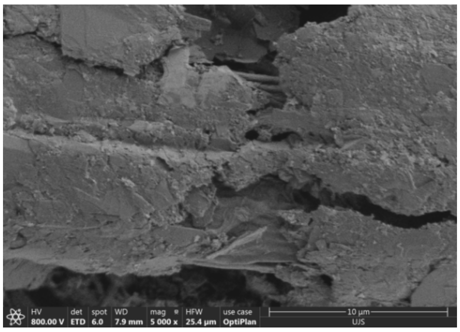

(**a**)

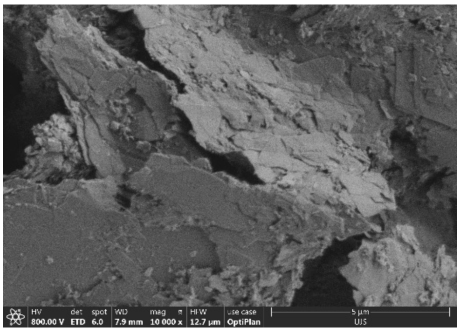

(**b**)

Figure S6. Image of functionalized graphene under a scanning electron microscope. (**a**) SEM images of the pencil-drawn graphite channel after functionalization at 10 μm scale. (**b**) SEM images of the pencil-drawn graphite channel after functionalization at 5 μm scale.

S3. Optimization of detection conditions for paper-based graphene-DNA1 sensors

S3.1 Effect of UP water on graphene sensor conductivity

The effect of ultrapure (UP) water on the conductivity of the graphene sensor was investigated by immersing the prepared sensor in UP water and monitoring the current at 30-min intervals. As depicted in Figure S7, the sensor current exhibited a gradual decline with increasing immersion time and reached a plateau after approximately 60 min. This observation indicates that prolonged exposure to an aqueous environment can alter the conductivity of the graphene sensor, likely attributable to hydration-induced structural or interfacial modifications. These findings highlight the importance of considering such environmental exposure when optimizing sensor design and operational protocols for enhanced long-term stability in practical applications.

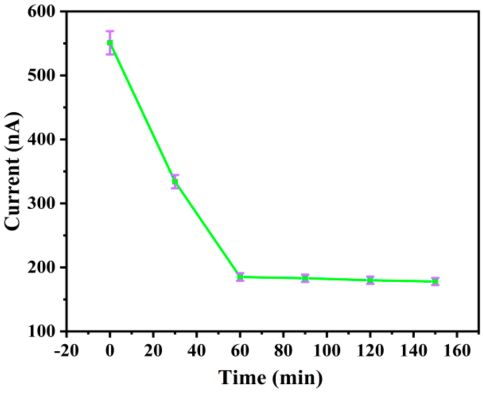

Figure S7. Effect of UP water on the conductivity of the graphene sensor.

S3.2 Optimization of detection frequency and voltage

The electrical characterization of the fabricated graphene–DNA1 sensor was performed using a lock-in amplifier integrated with a probe station. A constant source–drain bias of 150.0 mV was applied while maintaining the gate bias at 0 V. To identify the optimal detection frequency, the sensor response was systematically evaluated across a frequency spectrum ranging from 10.0 Hz to 50.0 kHz, including intervals at 10.0 Hz, 20.0 Hz, 30.0 Hz, 40.0 Hz, 50.0 Hz, 60.0 Hz, 70.0 Hz, 80.0 Hz, 90.0 Hz, 100.0 Hz, 200.0 Hz, 300.0 Hz, 400.0 Hz, 500.0 Hz, 600.0 Hz, 700.0 Hz, 800.0 Hz, 900.0 Hz, 1.0 kHz, 5.0 kHz, 10.0 kHz,

15.0 kHz, 20.0 kHz, 25.0 kHz, 30.0 kHz, 35.0 kHz, 40.0 kHz, 45.0 kHz, and 50.0 kHz. At each frequency, both the signal and background currents were recorded, and the corresponding signal-to-noise ratio (SNR) was calculated. As illustrated in Figure S8, the SNR increased with frequency and reached a plateau at approximately 35.0 kHz, beyond which further frequency increases yielded negligible improvement in SNR. Therefore, 35.0 kHz was selected as the optimum detection frequency for all subsequent current measurements.

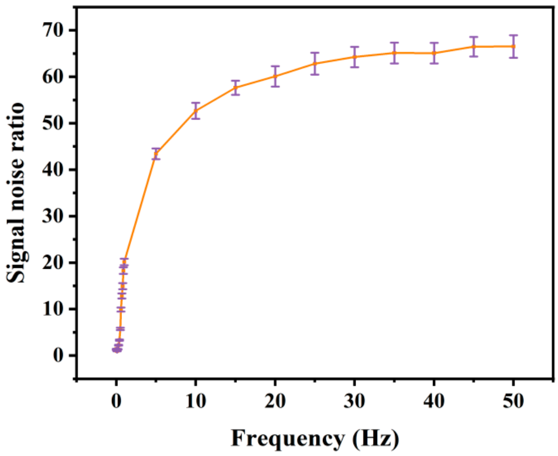

Figure S8. Signal-to-noise ratio with different frequencies.

To determine the optimal source–drain bias for current measurement, the gate bias was maintained at 0 V, and the detection frequency was fixed at 35.0 kHz. The source–drain bias was systematically varied from 20.0 mV to 200.0 mV in 20.0 mV increments (20.0 mV, 40.0 mV, 60.0 mV, 80.0 mV, 100.0 mV, 120.0 mV, 140.0 mV, 160.0 mV, 180.0 mV, and 200.0 mV), and the resulting current was recorded. As shown in Figure S9, the measured current displayed an approximately linear dependence on the applied source–drain bias, indicating stable ohmic contact and consistent device performance. Based on this linear response and overall signal stability, a source–drain bias of 100.0 mV was selected for all subsequent electrical measurements.

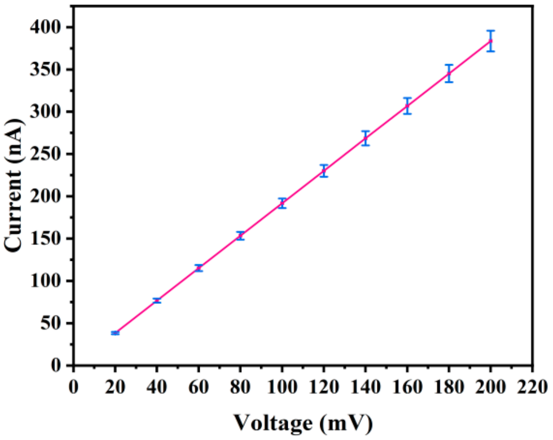

Figure S9. Current under different voltages between source and drain.

S3.3 Optimization of DAN concentration

To establish optimal detection conditions, the concentration of 1,5-diaminonaphthalene (DAN) was first optimized. The baseline current (I_0) of the fabricated graphene device was measured using a lock-in amplifier. Subsequently, methanol solutions containing

different concentrations of DAN (5 μM, 10 μM, 15 μM, 20 μM, 25 μM, and 30 μM) were deposited onto the graphene surface and incubated at room temperature for 3 h, followed by drying. Then, a 2% glutaraldehyde (GA) solution was applied and allowed to react under the same conditions for another 3 h before drying. Finally, a 20 nM DNA1 solution was introduced and incubated at 4 °C for 6 h. After drying, the resulting current (I) was measured again under identical electrical settings. As shown in Figure S10, the normalized current change ($\Delta I = I_0 - I$) reached a maximum at a DAN concentration of 15 μM, indicating that this concentration produced the most pronounced modulation of the sensor signal. Therefore, a DAN concentration of 15 μM was selected for all subsequent experiments to ensure optimal sensor performance.

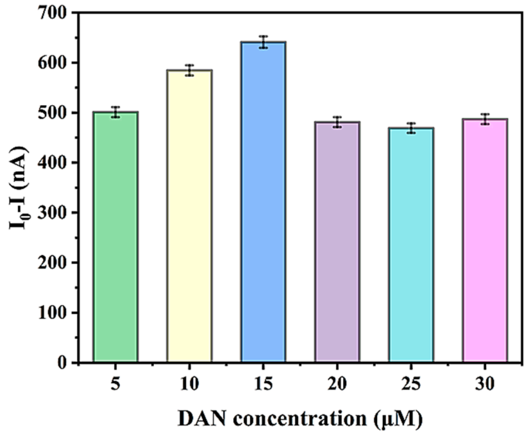

Figure S10. Optimization results of DAN concentration.

S3.4 Optimization of DNA concentration

The DNA1 concentration was optimized by evaluating a series of concentrations ranging from 10 nM to 70 nM (10 nM, 20 nM, 30 nM, 40 nM, 50 nM, 60 nM, and 70 nM). Each concentration was applied to graphene devices previously functionalized with DAN and GA, followed by incubation at 4 °C for 6 h and drying. The resulting current (I) was then measured using a lock-in amplifier. As shown in Figure S11, the measured current gradually decreased with increasing DNA1 concentration until reaching a plateau at approximately 40 nM, beyond which further increases yielded minimal additional change. To ensure both efficient use of materials and optimal sensor response, a DNA1 concentration of 40 nM was selected for all subsequent experiments.

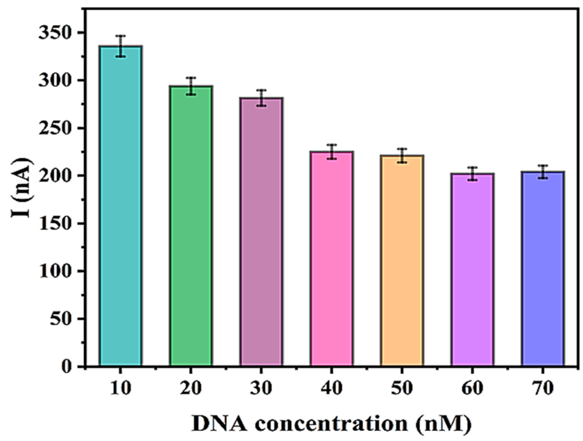

Figure S11. Optimization results of DNA1 concentration.

https://doi.org/10.3390/xxxxx

S3.5 Optimization of Hg^{2+} reaction time

To determine the optimal Hg^{2+}-DNA1 interaction time, the graphene–DNA1 sensor was first prepared by immobilizing a 40 nM DNA1 solution on the functionalized graphene substrate at 4 °C for 6 h, followed by drying. The initial baseline current (I_0) was recorded. Subsequently, 40 μL of a 10 nM Hg^{2+} solution was applied to the sensor surface, and the current (I) was measured at successive time intervals. As shown in Figure S12, the current initially remained above I_0 for up to 27 min, then decreased below I_0 after 28 min, and continued to decline gradually thereafter. The signal stabilized after approximately 1 h, indicating that the binding reaction had reached equilibrium. Therefore, a reaction time of 1 h was selected for all subsequent experiments to ensure consistent and complete Hg^{2+} detection.

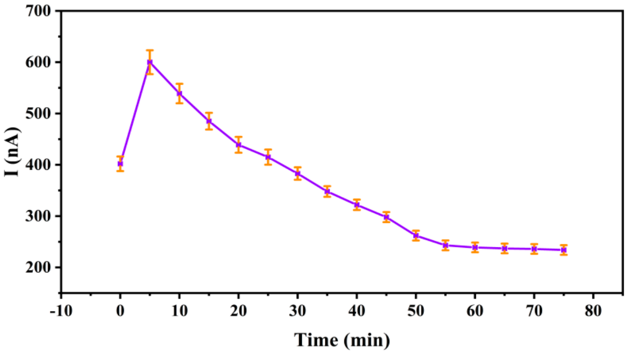

Figure S12. Optimization results of Hg^{2+} reaction time.

S3.6 Temperature experiments of graphene-DNA1 sensor

To validate the practical applicability of the paper-based graphene–DNA1 sensor, its performance was evaluated across a range of temperatures. The sensor response to a fixed concentration of Hg^{2+} was measured at temperatures varying from 10 °C to 70 °C. The initial current (I_0) of the prepared graphene-DNA1 sensor was measured using a lock-in amplifier. Subsequently, 30 μL of a 1000 pM Hg^{2+} solution was introduced onto the sensor surface and incubated at varying temperatures ranging from 10 °C to 70 °C for 1 h, before measuring the resultant current (I).

As shown in Figure S13, the sensor exhibited stable and consistent signal output between 10 °C and 60 °C, indicating robust operational stability under varied thermal conditions. A noticeable decrease in response was observed at 70 °C, likely attributable to thermal denaturation of the DNA probe or alterations in interfacial interactions at elevated temperatures. These results confirm the reliable functionality of the sensor across a broad temperature range relevant to environmental and analytical applications.

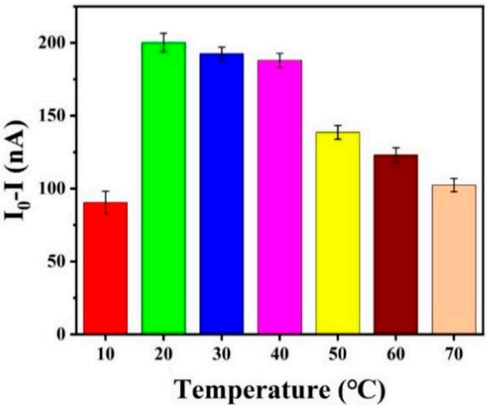

Figure S13. Responses of graphene-DNA1 sensor at different temperatures.

Figure S13 indicates that the sensor exhibits optimal performance at approximately 20 °C. When the temperature exceeds 70 °C, the activity of both glutaraldehyde (GA) and the DNA probe is compromised due to thermal denaturation, leading to a loss of structural integrity and functionality, thereby rendering the sensor ineffective.

S3.7 pH value experiments of graphene-DNA1 sensor

pH is a critical parameter influencing the performance of sensors in aqueous media. To evaluate the pH resilience of the graphene–DNA1 sensor, its response to Hg^{2+} was characterized across a broad pH range from 1 to 14.

The initial current (I_0) of the prepared graphene–DNA1 sensor was measured using a lock-in amplifier. A 30 μL aliquot of 1000 pM Hg^{2+} solution was then introduced onto the sensor surface and incubated for 1 h across a pH series ranging from 1 to 14. Following incubation, the resultant current (I) was measured again.

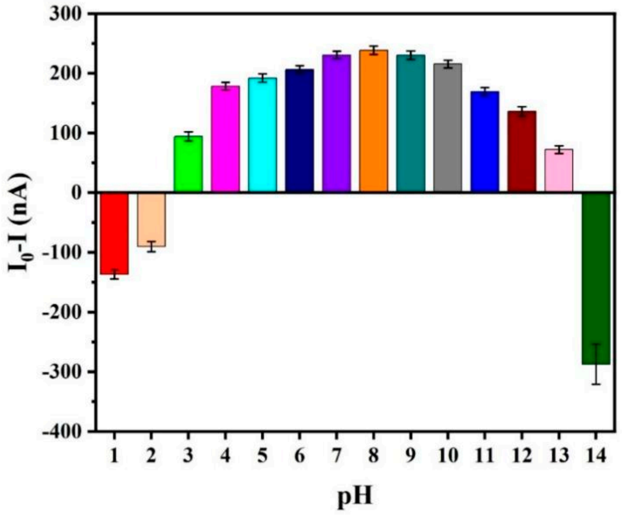

Figure S14. Responses of graphene-DNA1 sensor at different pH values.

Figure S14. demonstrates that the sensor maintains reliable detection capability within a pH range of approximately 6 to 10. It exhibits optimal performance at pH 8, achieving maximum detection efficiency. However, under strongly acidic (pH < 6) or strongly alkaline (pH > 10) conditions, the sensor's functionality is significantly compromised, likely due to the destabilization of the DNA probe structure or adverse effects on the interfacial chemistry essential for signal transduction.

S4. Calculation of LOD values

Formula:

$$S_{y/x} = \sqrt{\frac{\text{Sum of Squares of Residuals}}{\text{Degrees of Freedom}}} \quad (2)$$

$$\text{LOD} = \frac{3.3 \times S_{y/x}}{b} \quad (3)$$

$S_{y/x}$: Standard error of regression (also called residual standard error);

Sum of Squares of Residuals: Sum of squared deviations between measured and predicted values;

Degrees of Freedom: Number of data points n minus number of parameters in regression equation (n-2 for linear regression).

S5. Sensitivity analysis

S5.1 Sensitivity analyses of graphene-DNA2 sensors

Figure S15 shows the sensitivity analyses of Hg^{2+} based on graphene-DNA2 sensors. When the concentration of Hg^{2+} was in the range of 5-50 pM, there was a clear linear relationship between the current change and it. The linear equation was y=0.0027x+0.2698, R^2=0.997, and the detection limit was 0.875 pM.

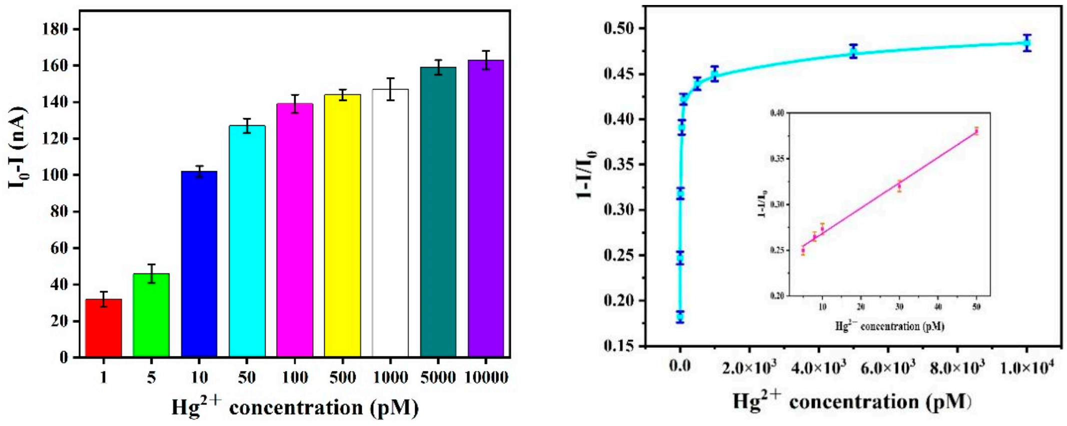

(**a**) (**b**)

Figure S15. (**a**) Sensitivity analyses of Hg^{2+} based on graphene-DNA2 sensors. (**b**) Current inhibition rate as a function of Hg^{2+} concentration based on graphene-DNA2 sensors.

S5.2 Sensitivity analyses of graphene-DNA4 sensors

Figure S16 shows the sensitivity analyses of Hg^{2+} based on graphene-DNA4 sensors. When the concentration of Hg^{2+} was in the range of 10-100 pM, there was a clear linear relationship between the current change and it. The linear equation was y=0.00182x+0.055, R^2=0.995, and the detection limit was 2.66 pM.

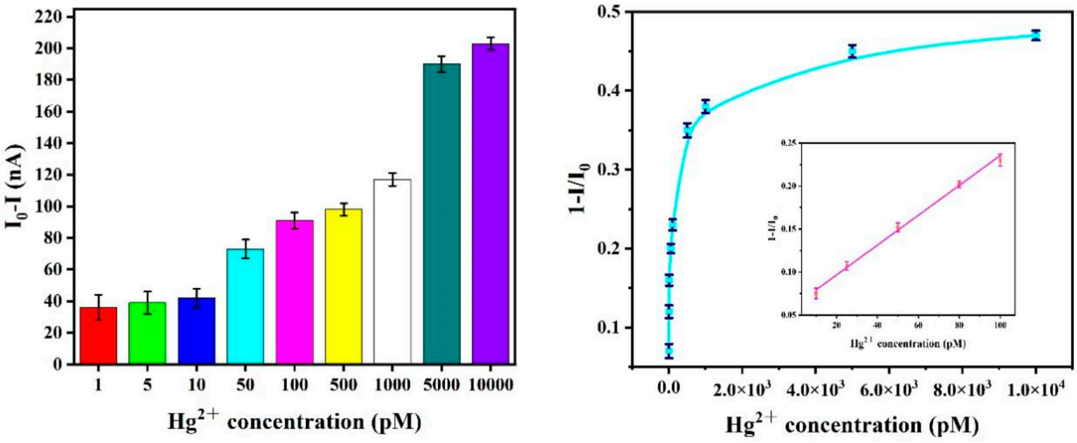

(**a**) (**b**)

Figure S16. (**a**) Sensitivity analyses of Hg^{2+} based on graphene-DNA4 sensors. (**b**) Current inhibition rate as a function of Hg^{2+} concentration based on graphene-DNA4 sensors.

S5.2 Sensitivity analyses of graphene-DNA5 sensors

Figure S17 shows the sensitivity analyses of Hg^{2+} based on graphene-DNA5 sensors. When the concentration of Hg^{2+} was in the range of 10-100 pM, there was a clear linear relationship between the current change and it. The linear equation was y=0.00117x+0.18865, R^2=0.996, and the detection limit was 3.78 pM.

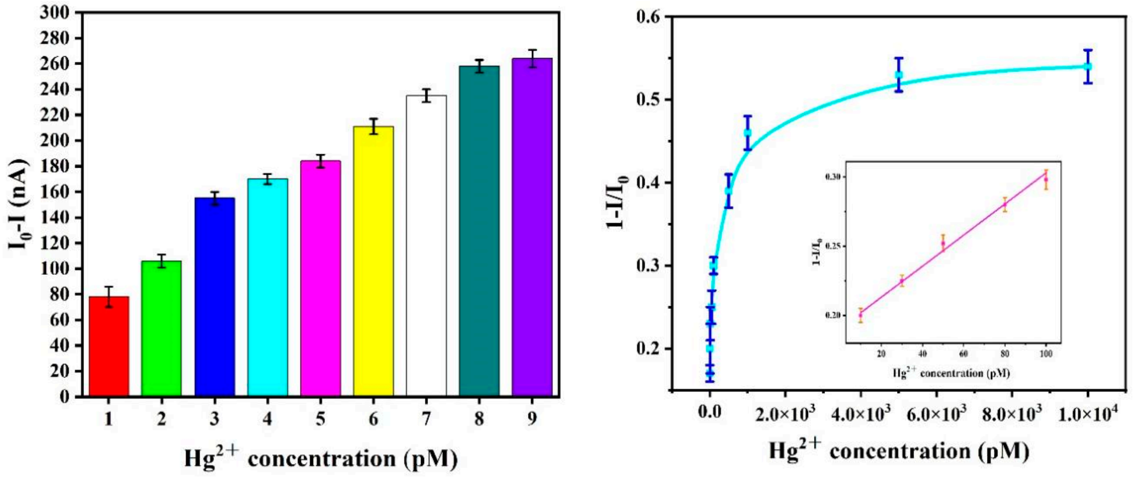

(**a**) (**b**)

Figure S17. (**a**) Sensitivity analyses of Hg^{2+} based on graphene-DNA5 sensors. (**b**) Current inhibition rate as a function of Hg^{2+} concentration based on graphene-DNA5 sensors.

S6 Characterization of the Binding of DNA with Mercury Ions

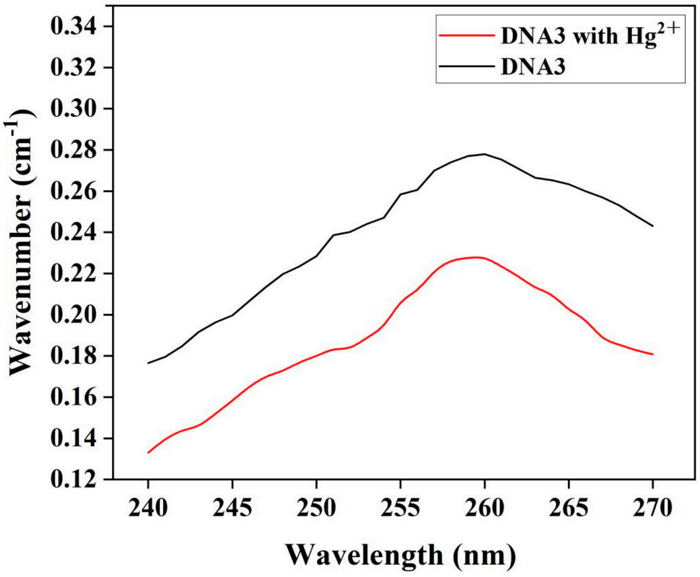

Figure S18. Absorbance of DNA3 and DNA3 with Hg^{2+}.
